# Development of Transdermal Oleogel Containing Olmesartan Medoxomil: Statistical Optimization and Pharmacological Evaluation

**DOI:** 10.3390/pharmaceutics15041083

**Published:** 2023-03-28

**Authors:** Rania Moataz El-Dahmy, Ibrahim Elsayed, Jihan Hussein, Mohammad Althubiti, Riyad A. Almaimani, Mahmoud Zaki El-Readi, Marawan A. Elbaset, Bassant M. M. Ibrahim

**Affiliations:** 1 Department of Pharmaceutics and Industrial Pharmacy, Faculty of Pharmacy, October 6 University, Central Axis, Cairo 12585, Egypt; 2 Department of Pharmaceutics and Industrial Pharmacy, Faculty of Pharmacy, Cairo University, Cairo 11562, Egypt; 3 Department of Pharmaceutical Sciences, College of Pharmacy, Gulf Medical University, Ajman 04184, United Arab Emirates; 4 Medical Biochemistry Department, Medicine and Clinical Studies Research Institute, National Research Centre, Giza 12622, Egypt; 5 Department of Biochemistry, Faculty of Medicine, Umm Al-Qura University, Al Abdeyah, Makkah 24381, Saudi Arabia; 6 Biochemistry Department, Faculty of Pharmacy, Al-Azhar University, Assuit 71524, Egypt; 7 Pharmacology Department, Medicine and Clinical Studies Research Institute, National Research Centre, Giza 12622, Egypt

**Keywords:** oleogel, olmesartan medoxomil, central composite design, texture analysis, ex vivo permeation, pharmacodynamic study, pharmacokinetic study

## Abstract

Olmesartan medoxomil (OLM) is a first-line antihypertensive drug with low oral bioavailability (28.6%). This study aimed to develop oleogel formulations to decrease OLM side effects and boost its therapeutic efficacy and bioavailability. OLM oleogel formulations were composed of Tween 20, Aerosil 200, and lavender oil. A central composite response surface design chose the optimized formulation, containing Oil/Surfactant (SAA) ratio of 1:1 and Aerosil % of 10.55%, after showing the lowest firmness and compressibility, and the highest viscosity, adhesiveness, and bioadhesive properties (Fmax and Wad). The optimized oleogel increased OLM release by 4.21 and 4.97 folds than the drug suspension and gel, respectively. The optimized oleogel formulation increased OLM permeation by 5.62 and 7.23 folds than the drug suspension and gel, respectively. The pharmacodynamic study revealed the superiority of the optimized formulation in maintaining normal blood pressure and heart rate for 24 h. The biochemical analysis revealed that the optimized oleogel achieved the best serum electrolyte balance profile, preventing OLM-induced tachycardia. The pharmacokinetic study showed that the optimized oleogel increased OLM’s bioavailability by more than 4.5- and 2.5-folds compared to the standard gel and the oral market tablet, respectively. These results confirmed the success of oleogel formulations in the transdermal delivery of OLM.

## 1. Introduction

Olmesartan medoxomil (OLM) is a potent first-line antihypertensive drug as it is a selective angiotensin II receptor blocker. It has few side effects such as tachycardia [1]. OLM is classified as BCS class II; it has a half-life of 10–15 h, log partition coefficient of 4.7, and molecular weight of 446.5 Da [2]. OLM is a prodrug that is converted rapidly to its active metabolite, Olmesartan, by undergoing de-esterification during its absorption from the gastrointestinal tract [3]. OLM has low oral bioavailability (28.6%) due to having poor water solubility and oral problems such as the extensive hepatic first-pass effect and the efflux pumps in the gastrointestinal tract that interfere with the drug’s absorption [3,4]. Thus, the transdermal drug delivery system (TDDS) is a recommended alternative technique to avoid the problems of OLM oral delivery and boost its therapeutic efficacy and bioavailability. TDDS can also sustainably deliver the drug to the systemic circulation, decreasing the dose frequencies and the drug side effects. Moreover, TDDS can de-esterify OLM and convert it to its active metabolite as a result of the presence of skin esterases [3].

Different researchers studied the transdermal delivery of OLM. In 2012, Hathout and Elshafeey formulated a transdermal microemulsion loaded with OLM. They stated that after implementing the clinical study in healthy human subjects, the relative bioavailability of the optimal microemulsion was 236.25% compared to the market tablet [3]. In 2016, Kamran et al. formulated a transdermal invasomal gel loaded with OLM. They reported that after implanting the gel in an in vivo study in Wistar rats, the optimal invasomal gel achieved a 1.15-fold increase in OLM bioavailability compared with the OLM market tablet [2]. In 2019, Albash et al. formulated transdermal bilosomes and transethosomes loaded with OLM and reported that upon in vivo pharmacodynamic investigations in Wistar rats, the optimal transdermal formulations significantly lowered the blood pressure to the normal values much better than the market tablet. Moreover, upon the in vivo kinetic study in albino rabbits, the transdermal optimal bilosomes and transethosomes formulations were found to significantly increase OLM bioavailability by more than 2- and 3-folds than the oral tablet, respectively [5,6].

So far, there is no published study about prepared oleogels to boost the transdermal delivery of OLM. Oleogels could offer various advantages over the gel preparations mentioned above in the previous studies, such as the easiness of oleogels preparation method and their long-term stability [7]. Additionally, oleogels have a unique structure and a lipophilic nature that can strongly boost the drug penetration through the stratum, delivering the drug to the bloodstream. [8]. Oleogels ensure better solubility of the hydrophobic drug and possess favorable rheological properties [9]. Moreover, they are resistant to microbial contamination because of the absence of any water, so they do not need the addition of preservatives [7]. Therefore, this study aimed to formulate OLM-loaded oleogel formulations to investigate their ability to enhance OLM solubility and to evaluate their safety, antihypertensive activity, and bioavailability-boosting capacity in experimental animals.

Oleogel, also called organogel, is a semisolid formulation developed by the gelation of oils using suitable gelling agents called organogelators [9]. These gelling agents form aggregates linked with weak interactions such as hydrogen bonds or Van der Waals forces, resulting in the formation of three-dimensional networks [10].

Our study used Aerosil 200, also known as fumed silica or colloidal silicon dioxide, as a gelling agent. Aerosil-based gels are stable over a wide temperature range and characterized by high viscosity and pronounced thixotropic behavior [11]. This is because of forming hydrogen bonds between the silica particles and between the silica particles and the oils, forming a three-dimensional network and developing a paste-like consistency [11,12].

Lavender oil, which is a natural essential oil, was chosen in this study due to having many advantages such as its excellent solubilizing power to OLM and its significant penetration enhancing effect. [13,14]. Moreover, lavender oil has a synergistic antihypertensive effect when combined with OLM. The exerted hypotensive impact of lavender oil could be attributed to increasing the serum nitrite levels and endothelium-independent vasorelaxation [1]. Lavender oil also decreases the common side effect of OLM (tachycardia). The exerted decreasing HR effect of lavender oil could be attributed to its positive impact on the autonomic nervous system, preventing OLM-induced increase in heart rate [1].

Tween 20 was used in this study as a surfactant due to its relatively low toxicity and great solubilizing power on hydrophobic drugs, such as OLM [15,16]. Additionally, Tween 20 is a potent permeation enhancer as it interacts not only with the biomembrane’s proteins but also with the membrane phospholipids by modifying the stratum corneum fluidity and permeability [17,18,19].

This study aimed to determine the prospective of oleogel formulations to augment OLM transdermal permeability, boost its bioavailability, maximize its therapeutic benefits, and decrease its side effects. The prepared oleogel formulations were investigated for their in vitro characterization, texture analysis, and ex vivo skin bioadhesive properties. Central composite response surface statistical design was utilized to obtain the optimal oleogel formulation employing Design Expert^®^ software version 7. Furthermore, in vitro drug release and ex vivo permeation studies were implemented for the optimized oleogel formulation compared to the drug suspension and gel. Moreover, in vivo histopathology, pharmacodynamic, and pharmacokinetic studies for OLM optimized oleogel formulation were performed upon comparison with OLM market tablet in female Sprague–Dawley rats.

## 2. Materials and Method

### 2.1. Materials

Olmesartan medoxomil was supplied by MultiApex Pharmaceutical Co. (Cairo, Egypt). Olmesartan (the active metabolite) was purchased from Santa Cruz Biotechnology Inc. (Santa Cruz, CA, USA). Lavender oil was from Pharmachem Pvt Ltd., Co. (Maharashtra, Mumbai, India). Tween 20, Aerosil 200, carboxy methylcellulose (CMC), Dexamethasone, and Fructose were from Sigma–Aldrich Co. (St. Louis, MO, USA). HPLC grade dexamethasone, triethylamine, ammonium acetate, acetonitrile, and methanol were purchased from Merck-Schuchardt Co. (Hohenbrunn, Bavaria, Germany). Angiosartan^®^ 10 mg oral tablet was from Chemiphar Co. (Cairo, Egypt).

### 2.2. Solubility Study

The solubility of OLM in different surfactants (Tween 20, Tween 80, Koliphor EL, and Koliphor RH 40) and different oils (lavender oil, olive oil, eucalyptus oil, and coconut oil) was assessed by utilizing the shake-flask technique [8]. In brief, excess amounts of OLM (50 mg) were added to different vials that contained 1 mL of the investigated surfactants or oils. Vials were set in a horizontal water bath shaker (PLT-110, Hyderabad, India) at 25 ± 0.5 °C for 24 h. The resulting mixtures were centrifuged at 3500 rpm for 15 min. The withdrawn supernatants were filtered using a membrane filter (0.45 µm), diluted with methanol, and then analyzed utilizing a spectrophotometer (Shimadzu, Kyoto, Japan) at λ_max_ 257 nm [5].

### 2.3. Oleogel Formulations Preparation

The surfactant and oil that showed maximum solubility of OLM were used with different ratios for preparing the oleogel formulations, as displayed in Table 1. In brief, lavender oil was heated on a magnetic stirrer (Heidolph Instruments, Schabach, Germany) at 60 °C, followed by adding Tween 20 with contentious stirring at 600 rpm. After that, 10 mg of OLM was added to with continuous stirring till the drug was completely dissolved. Then, Aerosil 200 (the gelling agent) was dispersed onto the obtained mixture. After the gelling agent was utterly blended and the mixture became homogenous, heating was stopped. The obtained mixture was allowed to cool down to gradually solidify and form the oleogel the room temperature [8].

### 2.4. Statistical Design

A central composite response surface design was implemented to investigate the impact of the different factors on the characteristics of the prepared oleogel formulations utilizing Design-Expert software version 7 (Stat-Ease Inc., Minneapolis, MN, USA). The Oil/SAA ratio (X_1_) and Aerosil % (*w/v*) (X_2_) were the two studied factors in this design: The elected responses were Viscosity (Y_1_), Firmness (Y_2_), Compressibility (Y_3_), Adhesiveness (Y_4_), Maximum detachment force (Fmax) (Y_5_), and Work of adhesion (Wad) (Y_6_). This design included nine formulations that had been generated with eleven runs, as formulation F5 was prepared three times to estimate the results’ repeatability. The composition of all the prepared oleogel formulations is displayed in Table 1. The optimized oleogel formulation was selected after having the highest desirability value that was obtained by showing the lowest firmness and compressibility, and the highest viscosity, adhesiveness (as absolute value), Fmax, and Wad.

### 2.5. Characterization of the Prepared Oleogel Formulations

#### 2.5.1. pH Determination

A pH meter (Thermo Scientific, Waltham, MA, USA) was used to detect the pH of OLM oleogel formulations. Prior to usage, the pH meter was calibrated utilizing a standard buffer solution. the study was carried out three times. Then, the mean value ± SD was estimated [8].

#### 2.5.2. Drug Content

Drug content test was performed to ensure the uniform distribution of OLM in the prepared oleogel preparations. One gram of each prepared oleogel formulation was dissolved first in methanol. Then, samples from the obtained liquids were diluted appropriately and analyzed spectrophotometrically at 257 nm [2].

#### 2.5.3. Viscosity Measurement and Determination of Rheological Characteristics

The viscosity of the prepared oleogel formulations was detected utilizing a cone and plate viscometer (Brookfield, Middleborough, MA, USA) at 25 ± 1 °C utilizing a CP-40 type spindle. The viscosity of the oleogel formulations was recorded at 10 s^−1^ shear rate. Additionally, the attained shear rate values were plotted versus that of shear stress to obtain the rheograms. The shear rate was in the range of 7.5 to 187.5 s^−1^ in case of the low viscosity oleogel formulations and from 2 to 100 s^−1^ in case of the high viscosity oleogel formulations [9,10].

#### 2.5.4. Texture Analysis

Texture properties of the prepared oleogel formulations were studied utilizing the CT3 Texture Analyzer. A standard beaker filled with 30 g of each prepared oleogel formulation was placed directly below a solid cylindrical probe (type TA-AACC) with a 21 mm diameter [20]. This probe penetrated twice into a 5 mm depth within the samples, and then it was redrawn again to its starting position with a 2 mm/s fixed speed [21]. Subsequently, the obtained force–time plot was used to detect different texture parameters of firmness, compressibility, and adhesiveness [9,20,21]. The obtained results were recorded and analyzed using Texture ProCT Software 1.5 (Brookfield Engineering Laboratories). This test was carried out three times for each sample [22].

#### 2.5.5. Ex Vivo Skin Bioadhesive Properties

Assessment of the bioadhesive properties of the oleogel formulations was performed on the rat skin model using Texture Analyzer (CT3, Brookfield, Middleboro, MA, USA) [23].

After shaving the rat dorsal region (area 3.14 cm^2^), the excised skin was preserved frozen for not more than 4 weeks at −20 °C. Before the start of the experiment, the excised skin was thawed for 30 min in normal saline solution. Then, a cyanoacrylate glue was used to adhere the rat skin on the lower end of the cylindrical probe. A standard beaker containing a 0.5 g sample from each oleogel formulation was placed below the probe that descended with a 0.1 mm/s speed till touching the oleogel sample. Both the sample and the rat skin were kept in contact for 120 s [24]. After that, the probe was drawn upwards at the same speed until the skin detached from the sample [21]. Subsequently, the obtained force–time plot was used to determine the bioadhesive characteristics, represented as Fmax and Wad [9].

### 2.6. Characterization of the Optimized Oleogel Formulation

#### 2.6.1. In Vitro OLM Release Study

In vitro release of OLM from the optimized oleogel formulation was implemented by applying the membrane diffusion technique utilizing the United States Pharmacopeia dissolution apparatus (type II, Pharm Test, Hainburg, Germany) [25]. The receptor compartment contained 500 mL phosphate buffer (pH = 7.4). The donor compartment (glass cylinder) consisted of two open ends; one end was fixed to the apparatus shaft, whilst the other side was covered with the dialysis membrane (12–14 kDa molecular mass cut-off) that was previously soaked in the dissolution medium overnight [26]. A sample from the optimized oleogel formulation (containing 5 mg OLM) was put inside the donor compartment. The shaft was stirred at 50 rpm at 37 ± 0.5 °C. In vitro release of OLM from both the drug suspension and gel was implemented by applying the same conditions for comparison. A total of 1 mL was taken from the drug suspension (5 mg OLM/mL). The drug gel had a concentration of 5 mg OLM/mL containing 1% CMC [27]. A sample from the drug gel (equivalent to 5 mg OLM) was taken for comparison. Samples (3 ml) were withdrawn at 0.25, 0.5, 1, 2, 3, 4, 6, 8, 12, and 24 h time intervals. Each withdrawn sample was replaced with the utilized dissolution medium for maintaining the perfect sink condition [28]. The withdrawn samples were analyzed utilizing a spectrophotometer [29]. The released OLM was detected at λ_max_ 257 nm [6]. This study was repeated in triplicate; then, the OLM average release % ± SD was calculated. The release kinetics of OLM from the optimized oleogel formulation, the drug suspension, and the drug gel were analyzed via linear regression analysis [27]. Similarity factors (*f_2_*) and dissolution half-life (T_50%_) were assessed for comparison [30,31].

#### 2.6.2. Ex Vivo Permeation Study

Newly born rats (100 ± 20 g) were sacrificed; then their dorsal skin was removed and preserved frozen in normal saline at −20 °C for two weeks maximum [32]. Prior to starting the experiment, the frozen skin was thawed till reaching room temperature. This study was implemented utilizing the USP dissolution apparatus II by applying the same conditions utilized in the in vitro drug release study [33,34]. The newly born rat skin was fixed on the glass cylinder having a surface area of 4.908 cm^2^. Then, a sample from the optimized oleogel formulation (containing 5 mg OLM) was placed in the donor compartment on the center of the stratum corneum surface. OLM permeation from the drug suspension and gel was conducted under the same conditions for comparison. A total of 3 mL was withdrawn at 0.25, 0.5, 1, 2, 3, 4, 6, 8, 12, and 24 h time intervals. OLM permeated concentration was detected in the gathered samples utilizing a validated HPLC method, where OLM was separated using a Thermo^®^ C18 column (150 mm × 4.6 mm). The mobile phase consisted of a mixture of deionized water/acetonitrile/triethylamine (60:40:0.3 *v*/*v*/*v*). Phosphoric acid (18%) was utilized to adjust the pH at 6.3 [35]. The permeated OLM was detected using the HPLC method at λ_max_ 257 nm [6]. This study was conducted three times, and the OLM mean permeated values ± SD were calculated [36]. The maximum permeation flux at 24 h (J_max_) and the enhancement ratio (ER) were assessed utilizing the following equations [28,31]:

(1) $$J_{\max}=\frac{\mathrm{Amount}\;\mathrm{of}\;\mathrm{OLM}\;\mathrm{permeated}}{\mathrm{Time}\;\times \;\mathrm{Memberane}\;\mathrm{area}}$$

(2) $$\mathrm{ER}=\frac{J_{\max}\;\mathrm{of}\;\mathrm{the}\;\mathrm{optimized}\;\mathrm{oleogel}\;\mathrm{formulation}\;}{J_{\max}\;\mathrm{of}\;\mathrm{the}\;\mathrm{drug}\;\mathrm{suspension}\;\mathrm{or}\;\mathrm{gel}}\;$$

One-way ANOVA followed by the Fisher’s least significant difference test was used for detecting any significant differences in the estimated values (*p*-value < 0.05).

### 2.7. In Vivo Study

Seventy-five female Sprague–Dawley rats (175–200 g body weight) were utilized in the in vivo studies. Fifteen rats were utilized in the histopathological study, and sixty were utilized in the pharmacodynamic study, biochemical analysis, and pharmacokinetic studies. The protocol of the study was approved after being revised by the Ethics Committee of October 6 University with reference number (PRE-Ph-2112004). The rats were housed in stainless-steel cages at 23 ± 1 °C, 55% humidity, and artificial illumination of dark/light cycle (12 h each). Chow and standard water were provided, and the room was free from contamination. The rats were kept for a week before starting the experiments for adaptation [5]. Hair was removed by closely shaving the dorsal part of the rats’ trunk [6].

#### 2.7.1. Histopathological Study

Fifteen female Sprague–Dawley rats were utilized in the in vivo histopathological study. The rats were equally divided into three groups. Group I served as the control. On the other hand, rats in groups II and III were subjected to topical application of the optimized oleogel formulation and lavender oil, respectively. The treatments were applied to the previously shaved dorsal rat skin (approximately 4 cm^2^). After ending the treatment period (1 day), the rats were sacrificed via the decapitation method following anesthesia, and the skins were excised for histopathological examination. Skin specimens were put in 10% formol saline for 24 h. After that, skin specimens were cut into 4 mm thick, stained with eosin and hematoxylin (E&H), and examined under a light microscope (Axiostar Plus, Oberkochen, Germany).

#### 2.7.2. Pharmacodynamic Study

A pharmacodynamic study was implemented utilizing different drug concentrations to choose the best dose that gives the desired drug efficacy and minimizes the side effects. For the determination of systolic and diastolic blood pressures and heart rate, this study was implemented using the cuff tail method [37]. Sixty female Sprague–Dawley rats were classified equally into ten groups (Groups A–J). Group A was the negative control group (normal). Induction of hypertension in the other groups (groups B–J) was implemented by the administration of 20% fructose orally for three weeks and dual injection of dexamethasone subcutaneously (0.1 mg/kg) on the nineteenth and twenty first day to augment blood pressure elevation [38,39]. Group B was the hypertensive positive control (untreated) group that did not receive any further treatment. Groups (C–I) received same weight of topical treatments after shaving the hair on their dorsal area with a determined area of 4 cm^2^. Groups (C–E) received topical treatment of OLM optimized oleogel formulation with three different concentrations (F0.18, F0.36, F0.72 mg/0.25 mL). Groups (F–H) received topical treatment of a standard gel, which is a drug gel with different concentrations (S0.18, S0.36, S0.72 mg/0.25 mL) containing 1% CMC. Group (I) received topical treatment of plain lavender oil (0.25 mL), while group (J) orally received the market tablet (1.8 mg/kg, which equals 0.36 mg/0.25 mL). The market tablet was mashed and given to the rats with the aid of oral gavage [40]. Then, the BP from the tail and the heart rate were recorded at a basal time before the induction of hypertension (zero time), after 3 weeks of hypertension induction, and after 1, 2, and 24 h of treatment. Statistical significance was performed using the One-Way ANOVA test utilizing SPSS^®^ software (version 22). Difference at *p* < 0.05 was considered significant. Graph pad prism software (version 5) was utilized for performing all the statistical tests.

After choosing the best dose that provided the desired efficacy, it was chosen for further biochemical and pharmacokinetic analysis.

#### 2.7.3. Blood Sampling for Biochemistry

For determination of sodium (Na^+^) and potassium (K^+^) levels in serum, blood samples (0.5 mL) were taken after 48 h of treatment following the three weeks of hypertension induction. Blood samples were taken from the normal, positive control, the optimized oleogel (0.36 mg/0.25 mL), standard gel (0.36 mg/0.25 mL), lavender oil (0.25 mL), and market tablet (1.8 mg/kg equivalent to 0.36 mg/0.25 mL) groups.

Kits were purchased from Bio-Diagnostic Co., Giza, Egypt. Blood samples were taken from the veins’ retro-orbital plexus. Gathered blood samples were set at 37 °C for 10 min, then centrifuged at 3000 rpm at 4 °C for 10 min, and serum was separated [41]. Determination of the serum level of Na^+^ was performed according to the method of Trinder [42] and of K^+^ according to the method of Sunderman and Sunderman [43]. Statistical significance was performed using the One-Way ANOVA test utilizing SPSS^®^ software (version 22). Difference at *p* < 0.05 was considered significant.

#### 2.7.4. Pharmacokinetic Study

Blood samples were collected from the optimized oleogel (0.36 mg/0.25 mL), standard gel (0.36 mg/0.25 mL), and market tablet (1.8 mg/kg equivalent to 0.36 mg/0.25 mL) groups that exhibited the highest efficacy in lowering the elevated BP after the induction of hypertension. A total of 3 mL blood samples were taken from the veins’ retro-orbital plexus into heparinized Eppendorf tubes after 0, 1, 2, 4, 8, 24, and 48 h of treatment. Centrifugation of the gathered blood samples was performed at 5000 rpm for 15 min. The obtained plasma was stored at −70 °C till analysis using the validated HPLC method [2].

The utilized internal standard (IS) is Olmesartan, the active form of OLM, with a working concentration of 10 µg/mL.

The employed HPLC system (Shimadzu, Tokyo, Japan) was equipped with a pump (LC-20 AT), injector (Rheodyne 7125), UV detector (SPD-20A), and Eurospher100^−5^ C18 column (4.6 × 250 mm), 5 μm particle size. The isocratic mobile phase consisted of acetonitrile: 0.1 mL triethylamine mixture: 0.05 M ammonium acetate buffer, while adjusting the pH to 6.8. It was delivered with a flow rate of 1 mL/min. The detector was fixed at 257 nm [2].

Olmesartan concentration was plotted versus the time to obtain the peak plasma concentration (C_max_), time of reach the peak concentration (T_max_), mean residence time (MRT), elimination half-life (t_1/2_), elimination rate constant (K_e_), and area under the plasma concentration–time curves from time zero to the last time point and to infinity (AUC_0–48_ and AUC_0–∞_). These pharmacokinetic parameters were analyzed utilizing a non-compartmental model, applying Kinetica^®^ software (version 5). Comparison of the calculated pharmacokinetic parameters of the treatments was performed using the One-Way ANOVA test utilizing SPSS^®^ 22 software (IBM Corporation, Armonk, NY, USA), followed by Tukey Kramer’s multiple comparisons test. The difference was considered significant at *p*-value < 0.05.

## 3. Results and Discussion

### 3.1. Solubility Study

In the formulation of the oleogel loaded with OLM, choosing a suitable surfactant and oil has an essential role as it enhances the drug loading and solubility and influences the dosage form efficacy, drug release stability, and quality [16]. Among the oils investigated in this study, lavender oil showed the maximum solubilizing power of OLM. Therefore, it was chosen as the oil component. The solubility of OLM in lavender oil, olive oil, eucalyptus oil, and coconut oil was 30.66, 7.73, 5.69, and 1.89 mg/mL, respectively. This is in an agreement with the results obtained by Balakumar et al., who formulated a self-emulsifying drug delivery system for a hydrophobic drug. They stated that lavender oil exhibited the highest solubility of the investigated drug, more than many other tested oils [13].

On the other hand, among the surfactants investigated in this study, Tween^®^ 20 (HLB = 16.7) showed the highest solubilizing capacity of OLM, followed by Tween^®^ 80 (HLB = 15), Cremophor^®^ RH 40 (HLB = 14–15), and finally Cremophor^®^ EL (HLB = 13). The solubility of OLM in Tween^®^ 20, Tween^®^ 80, Cremophor^®^ RH 40, and Cremophor^®^ EL were 8.89, 6.57, 5.1, and 4.24 mg/mL, respectively. It is notable that the solubility of OLM was dependent on the HLB value of the utilized surfactants; a higher HLB value of the surfactant was associated with a higher OLM solubility [16].

### 3.2. Preparation of Oleogel Formulations Loaded with OLM

The preparation method succeeded in preparing the oleogel formulations loaded with OLM, where the prepared formulations were homogenous and clear. The chosen lavender oil and Tween 20 surfactant succeeded in solubilizing the drug efficiently in the prepared oleogels, where the drug completely disappeared in the prepared oleogels, and the resulted oleogel formulations were utterly transparent [13,16]. Aerosil 200 (fumed silica) successfully increased the viscosity of the oleogel formulations. The prepared oleogel formulations had an appropriate consistency that provided the desired spreadability of the oleogel for an extended period at the application site [8]. This effect is attributed to the formation of hydrogen bonds between the silica particles with each other and between the silica particles and the lavender oil, forming a three-dimensional network and developing the desired consistency [11,12].

### 3.3. Statistical Analysis of the Experimental Design

Central composite response surface statistical design was implemented using ANOVA. Adequate precision was used to ensure the convenience of the selected model in navigating the design space. Adequate precision values were higher than 4 in all the responses, as shown in Table 2, which was desirable. Moreover, the predicted R^2^ was in good harmony with the observed R^2^ in all the detected responses, indicating the chosen model’s suitability to predict the responses’ values. Table 2 displays all the output data of the statistical design. Differences at *p* ≤ 0.05 were considered significant.

### 3.4. Characterization of the Prepared Oleogel Formulations Loaded with OLM

#### 3.4.1. pH Determination

The pH of the semisolid formulations can affect the solubility and stability of the loaded drug and can influence its skin irritation potential. Thus, oleogel formulations should have pH values within the physiologically accepted range (4.5–6.5) to be safe and non-irritant. The pH values of all the prepared oleogel formulations were within the required range, where they were found to be from 4.91 ± 0.27 to 6.20 ± 0.31, as displayed in Table 1. Thus, the prepared oleogels can be applied safely on the skin without causing any irritation problems [44].

#### 3.4.2. Drug Content

The prepared oleogel formulations had drug content values within the accepted USP pharmacopeial limit (90%–110%) for the semisolid formulations [8,45]. The drug content varied from 94.86 ± 2.01% to 99.06 ± 4.95% of the labeled claim. These outcomes ensure the content uniformity and the homogenous OLM dispersion in all the prepared oleogel formulations.

#### 3.4.3. Viscosity Measurement and Rheological Characteristics Determination

Viscosity and rheological characteristics of the prepared oleogel formulations affect their application behavior on the skin and their contact time. They also may affect their mechanical proprieties (firmness, compressibility, and adhesiveness) and drug release. The viscosity of the prepared oleogel formulation should be high enough to impart the desired consistency that guarantees ease of administration and sufficient long contact time with the skin.

As displayed in Table 1, the viscosity of the prepared oleogel formulations ranged from 686.4 ± 13.90 to 48,620 ± 183.65 cps. The calculated equation for the viscosity values analysis was:

Viscosity = 17,859.26 + 9994.60 X_1_ + 15,715.52 X_2_ + 6184.60 X_1_·X_2_ (3)

**Figure 1:**
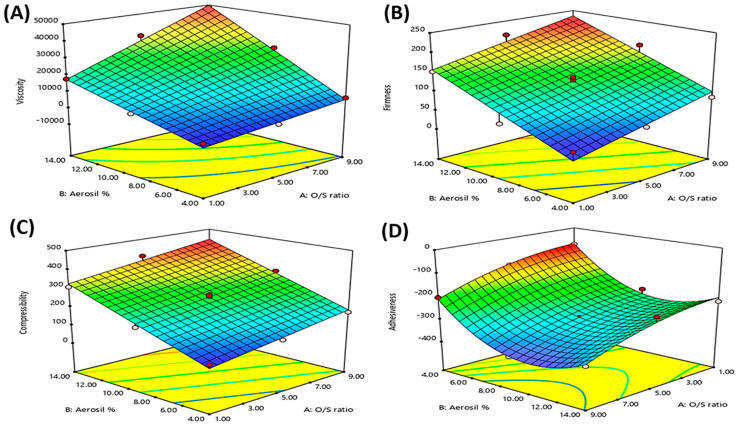
Response surface plots for the effects of O/SAA ratio (X_1_) and Aerosil % (X_2_) on the viscosity (**A**), firmness (**B**), compressibility (**C**), and adhesiveness (**D**) of OLM oleogel formulations.

Table 1 and Figure 1A clarify that the viscosity of all the prepared oleogel formulations was affected by both the independent variables, the Oil/SAA ratio (X_1_) and the Aerosil % (X_2_), where increasing both the independent variables significantly increased the viscosity of the prepared oleogel formulations. This could be attributed to that increasing the Oil/SAA ratio (increasing the oil amount) and the % of Aerosil increased the interaction between the oil and the Aerosil’s silanol group via hydrogen bonding, leading to the formation of a three-dimensional structure and providing the semisolid consistency [11,46].

Moreover, there was an interaction between the two factors (X_1_·X_2_) that significantly impacted the viscosity, where the highest viscosity values were obtained at high levels of both factors simultaneously, as illustrated in Supplementary Figure S1. On increasing the Oil: SAA ratio (X1), the viscosity was significantly increased at a high level of Aerosil % (X2) than at a low level of Aerosil % (X2).

**Figure 2:**
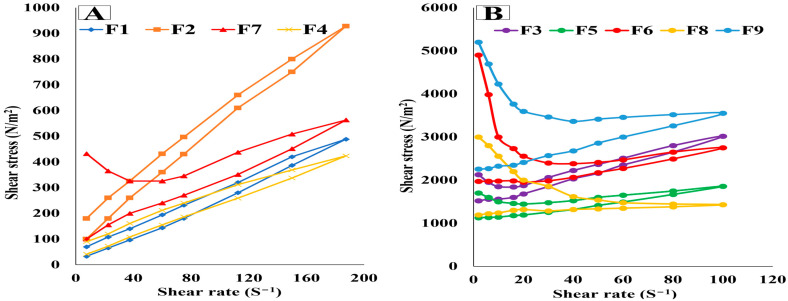
Rheograms showing the shear stress at different rates of shear for (**A**) low viscosity and (**B**) high viscosity oleogel formulations.

Figure 2 illustrates the rheograms of the prepared oleogel formulations, which deducted that all the oleogel formulations had a non-Newtonian pseudoplastic system with shear thinning flow. Furthermore, they showed thixotropic characteristics, as proved by the presence of the hysteresis loops on the rheograms, where viscosity decreased under shearing stress due to breaking down the hydrogen bonds between silica particles and the oil. During the absence of mechanical forces, hydrogen bonds reform again, rejoining the silica particles, rebuilding the three-dimensional network quickly, increasing the viscosity back to the initial value, and rapidly recovering the oleogel structure. This behavior is very desirable for the application of semisolid formulations, where it aids in forming a thin layer of the formulation upon its application on the skin and provides a long contact time, leading to efficient drug delivery at the application site [8]. These results are in harmony with that reported by Wróblewska et al., who formulated Ketoconazole oleogels containing Aerosil. He stated that Aerosil succeeded in formulating oleogels with a shear-thinning behavior and thixotropic characteristics that facilitated the application of the gel and increased its retention after application [10]. Similar findings were declared also by Lu and Fassihi who formulated Diclofenac gel containing Aerosil. They stated that the incorporation of Aerosil formulated a highly viscous gel with thixotropic properties, providing high spreadability and retention of the gel on the skin upon application, decreasing the application frequency, and increasing patient compliance [11].

#### 3.4.4. Texture Analysis

Texture profile analysis (TPA) is a useful technique in the pharmacy fields to characterize the semisolid dosage form, where it provides crucial information on the sample’s response to the external forces [47,48]. Moreover, TPA is very beneficial in confirming the spreadability of the semisolid formulations on the skin surface and their ease of removal from the container. This is in agreement with Coviello et al., who stated that the texture analysis is very important in the fields of pharmacy [49].

#### Firmness

Firmness is the maximum force required for attaining the sample deformation. The ideal semisolid preparations should possess low firmness to ensure their good spreadability at the application site and the easiness of their removal from the container [8].

As displayed in Table 1, the firmness of the prepared oleogels was found to be significantly different from each other, where they ranged from 42.3 ± 2.98 to 221.7 ± 7.64 N. The calculated equation for the firmness values analysis was:

Firmness = 127.98 + 39.55 X_1_ + 67.77 X_2_ (4)

Table 1 and Figure 1B show that both the independent variables, the Oil/SAA ratio (X_1_) and the Aerosil % (X_2_), significantly impacted the firmness of the prepared oleogel formulations. It is worth noting that the firmness significantly increased with increasing both the Oil/SAA ratio and the Aerosil %. This could be attributed to that increasing both the oil and the Aerosil resulted in increasing the formation of hydrogen bonding between the oil and the Aerosil’s silanol group, resulting in the three-dimensional structure formation and providing the semisolid consistency [11,12].

#### Compressibility

Compressibility can be defined as the work needed for deforming the sample during compressing the probe on it. The ideal semisolid preparations should possess low compressibility to ensure good spreadability and easy application of these semisolid preparations on the skin [8].

The compressibility of the prepared oleogels varied from 87.7 ± 4.40 to 427.6 ± 10.36 mJ, as displayed in Table 1. The calculated equation for the compressibility values analysis was:

Compressibility = 255.26 + 58.28 X_1_ + 128.53 X_2_ (5)

Table 1 and Figure 1C show that both the independent variables, the Oil/SAA ratio (X_1_) and the Aerosil % (X_2_), significantly impacted the compressibility of the prepared oleogel formulations. Notably, the compressibility was significantly increased with increasing the Oil/SAA ratio and the Aerosil %. This could be attributed to that increasing both the oil and the Aerosil % resulted in providing the semisolid consistency, as explained earlier [11,12].

#### Adhesiveness

Adhesiveness is the required work to remove the probe from the sample. In the case of semi-solid preparations, higher adhesiveness values are desired to ensure prolonged adhesion and residence of the semi-solid preparations at the application site [8,20].

The adhesiveness of the prepared oleogels varied from −76.2 ± 5.35 to −390.1 ± 11.82 mJ, as displayed in Table 1. The calculated equation for the adhesiveness values analysis was:

Adhesiveness = −295.39 − 66.83 X_1_ − 67.88 X_2_ + 2.03 X_1_·X_2_ − 27.33 X_1_^2^ + 112.72 X_2_^2^ (6)

The adhesiveness of the prepared oleogel formulations was impacted by both the independent variables, as displayed in Table 1 and Figure 1D. The adhesiveness was significantly increased with increasing the Oil/SAA ratio (X_1_) and the Aerosil % (X_2_). This could be due to that increasing the oil and the Aerosil % led to increasing the hydrogen bonds formation between the oil and the Aerosil, forming a more robust three-dimensional structure and a highly viscous adhesive gel [11,46].

On the other side, the further excessive increase in the Aerosil % (X_2_) resulted in decreasing the adhesiveness of the oleogels, where adhesiveness was reduced in oleogels with Aerosil % of 14% than in those with 9%. This could be attributed to the coating influence of Aerosil powder, where the excess amount of Aerosil powder coats the outer surface of the oleogel, providing a smooth and less adhesive surface, resulting in decreasing the stickiness or adhesiveness [50,51,52,53].

#### 3.4.5. Ex Vivo Skin Bioadhesive Properties

The bioadhesive phenomenon is advantageous for transdermal delivery, as it increases the contact time of the semisolid preparations on the skin, increasing the drug permeation through the skin. Moreover, the bioadhesive nature of the semisolid preparations decreases the treatment administration frequency and improves the patient’s compliance [23,24].

Bioadhesion means adherence of the synthetic or natural materials onto the biological surface. This strong adhesion happens when the material and the biological surface are capable of forming hydrogen, covalent, ionic, or polar bonds [21,54]. The ideal semisolid preparation should possess high values of detachment force (Fmax) and work of adhesion (Wad) to ensure its long contact time on the skin.

The Fmax of the prepared oleogels varied from 131.6 ± 4.79 to 539.7 ± 16.11 mN, while the Wad of the prepared oleogels varied from 176.4 ± 3.96 to 741.7 ± 20.48 µJ, as displayed in Table 1.

The following equations were used to analyze the Fmax and Wad values, respectively:

Fmax = 446.77 + 12.35 X_1_ + 108.48 X_2_ − 53.00 X_1_·X_2_ + 52.63 X_1_^2^ − 172.37 X_2_^2^ (7)

Wad = 500.75 − 48.62 X_1_ + 162.97 X_2_ − 76.63 X_1_·X_2_ + 130.98 X_1_^2^ − 206.37 X_2_^2^ (8)

**Figure 3:**
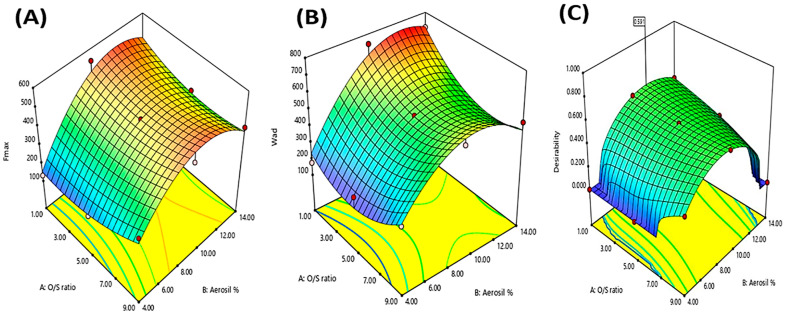
Response surface plots for the effects of O/SAA ratio (X_1_) and Aerosil % (X_2_) on the Fmax (**A**), Wad (**B**), and desirability (**C**) of OLM oleogel formulations.

Table 1 and Figure 3A,B show that the Fmax and Wad values of the prepared formulations were only affected by the Aerosil % (X_2_), respectively. Obviously, increasing the Aerosil % led to a significant increase in the Fmax and Wad values of the prepared oleogels, where oleogel formulation with Aerosil% (9%) showed much greater Fmax and Wad values than those containing (1%) Aerosil. These findings might be due to that increasing Aerosil % increased the hydrogen bonds formation between Aerosil’s silanol groups and the proteins present in the skin, resulting in increasing the bioadhesiveness [55].

On the other hand, a further increase in Aerosil % (X_2_) (14%) showed a decrease in the bioadhesive parameters (Fmax and Wad), where oleogel formulations with Aerosil % (14%) had lower Fmax and Wad values than oleogel formulations with lower Aerosil % (9%). Similar findings were declared by Lu and Fassihi and Wróblewska et al., who reported that the excessive increase in the Aerosil % means increasing the interaction between the oil and the Aerosil’s silanol groups, forming a more rigid three-dimensional structure that prevents the bonds’ formation between the applied gel and the proteins present in the biological membranes. Thus, this leads to decreasing the bioadhesive characteristics of the semisolid preparation [8,11].

In addition, there was an interaction between both the studied factors (X_1_·X_2_) that had a significant effect on the bioadhesive parameters (Fmax and Wad), where both Fmax and Wad were decreased only by increasing both factors simultaneously, as illustrated in the Supplementary Figures S2 and S3, respectively. At high Aerosil % (X_2_), increasing the Oil/SAA ratio (X_1_) decreased the bioadhesive parameters’ values, while at low Aerosil % (X_2_), increasing the Oil/SAA ratio (X_1_) did not reduce the bioadhesive parameters’ values (Fmax and Wad).

### 3.5. Selection of the Optimized Oleogel Formulation

The desirability value, estimated using the Design-Expert-7^®^ software, was utilized to choose the optimized oleogel formulation by determining the optimum composition that would yield all the desired responses. Figure 3C illustrated that the optimized oleogel formulation, containing Oil/SAA ratio (1:1) and Aerosil % 10.55%, showed the highest desirability value (0.591). This optimized oleogel formulation collectively had the highest viscosity, adhesiveness, Fmax, and Wad, and the lowest firmness and compressibility. It showed a viscosity of 10,769.4 ± 47.94 Cps, firmness of 113.19 ± 7.13 N, compressibility of 229.97 ± 9.46 mJ, adhesiveness of −251.63 ± 11.92 mJ, Fmax of 532.12 ± 18.44 mN, and Wad of 730.86 ± 23.08 µJ. Table 2 displays a high similarity between the observed and predicted values, ensuring the model performance accuracy [56].

### 3.6. Characterization of the Optimized Oleogel Formulation

#### 3.6.1. In Vitro OLM Release from the Optimized Oleogel Formulation

**Figure 4:**
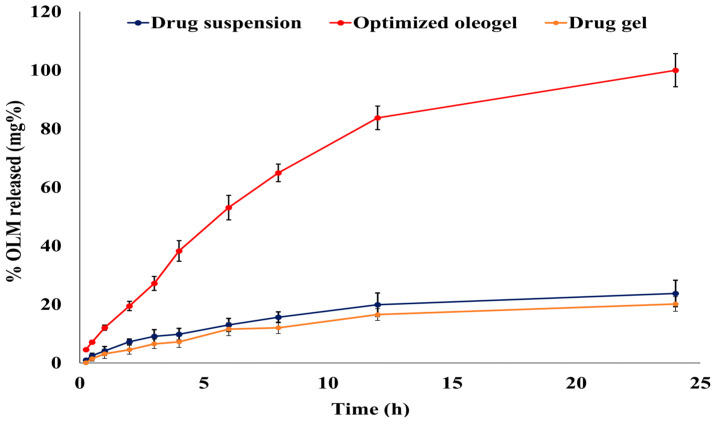
Release profiles of OLM from the optimized oleogel formulation compared to the drug suspension and gel in phosphate buffer (pH 7.4) at 37 °C. Presented data are the mean ± SD (n = 3).

The optimized oleogel formulation significantly increased the OLM dissolution rate and extent compared to both the drug suspension and gel, with *f2* values of 18 and 17, respectively [57]. The OLM optimized formulation, the drug suspension, and the drug gel followed the Korsmeyer-Peppas model with an anomalous release (R^2^ = 0.9936, 0.9874, and 0.9784, respectively). There was a significant difference between the release T_50%_ of the optimized OLM oleogel formulation (7.14 h) and the release T_50%_ of both the drug suspension (54.47 h) and the drug gel (62.12 h) (*p*-value < 0.001). As shown in Figure 4, the optimized oleogel released 100% of the drug after 24 h, while the drug suspension and gel released only 23.75% and 20.11% of OLM, respectively. Thus, the optimized oleogel formulation increased OLM release by 4.21- and 4.97-folds compared to the drug suspension and gel, respectively. This significant increase in OLM release from the optimized oleogel formulation could be attributed to its incorporation of Tween 20 and lavender oil, which have an excellent solubilizing power on the hydrophobic drugs, such as OLM, enhancing its diffusion and release from the optimized oleogel formulation to the dissolution medium [13,15].

#### 3.6.2. Ex Vivo Permeation Study

**Figure 5:**
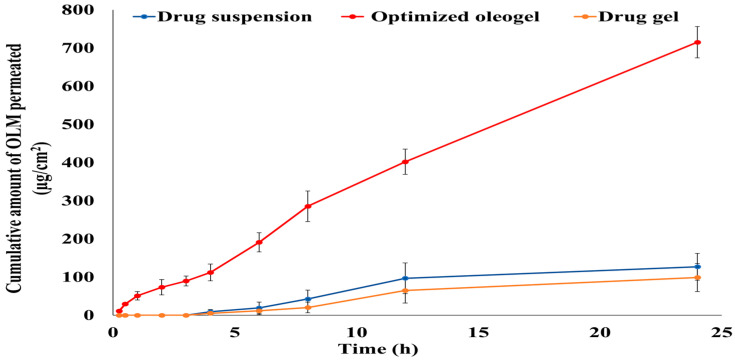
Ex vivo permeation profile of OLM from the optimized oleogel formulation in comparison with the drug suspension and gel through newly born rat skin. Presented data are the mean ± SD (n = 3).

Transdermal permeation of OLM from the optimized oleogel formulation was estimated through newly born rat skin. As illustrated in Figure 5, the optimized oleogel formulation significantly increased OLM permeation compared to the drug suspension and gel. After 24 h, 70.09% of OLM permeated from the optimized oleogel formulation, while just 12.6% and 9.6% permeated from the drug suspension and gel, respectively. The flux (J_max_) value of the optimized oleogel formulation (30.09 μg/h/cm^2^) was significantly different from the J_max_ values of the drug suspension (5.35 μg/h.cm^2^) and the drug gel (4.16 μg/h.cm^2^) (*p*-value < 0.001). Additionally, the enhancement ratio (ER) of the optimized oleogel formulation was 5.62 and 7.23 upon comparison with the drug suspension and gel, respectively, indicating increasing OLM permeation from the optimized formulation by more than 5 and 7 folds than the drug suspension and gel, respectively. This significant increase in OLM permeation from the optimized formulation could be attributed to its incorporation of Tween 20, which had great solubilizing power on the drug. This could also be due to the vital role of tween 20 as a permeation enhancer that interacts not only with the biomembrane’s proteins but also with the membrane phospholipids by modifying the stratum corneum fluidity and permeability [17,18,19]. Additionally, these findings could be attributed to the impact of lavender oil (essential oil) as a permeation enhancer in enhancing OLM permeation through the rat skin [14].

The significant low permeability of the drug suspension and gel could be attributed to the drug’s characteristics, where the poor OLM permeation could be due to the drug’s high log partition coefficient value (log p 5.6) [58]. There is a distinguishable parabolic relationship between the drug’s lipophilicity and its ability to permeate the skin, with the highest power to permeate at log P of approximately 3 to 4 [59].

### 3.7. In Vivo Studies

#### 3.7.1. Histopathological Study

**Figure 6:**
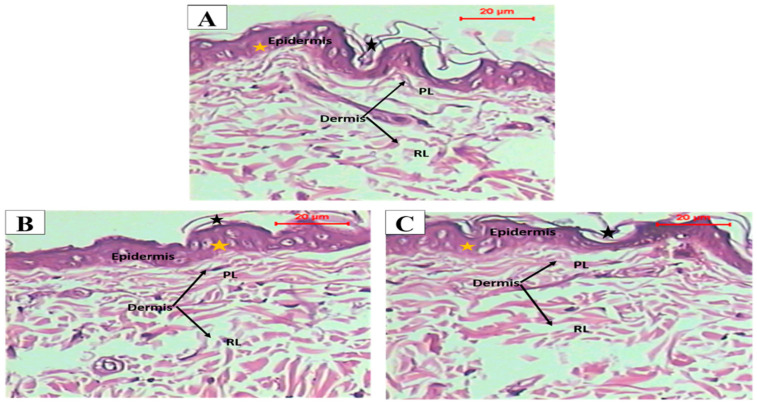
Photomicrographs showing the histopathological sections of (**A**) normal untreated rat skin, (**B**) rat skin treated with OLM optimized oleogel formulation, and (**C**) rat skin treated with lavender oil with magnification power of ×200 to illustrate the epidermis and dermis layers. Abbreviations: PL, papillary layer and RL, deeper reticular dermal layer.

**Figure 7:**
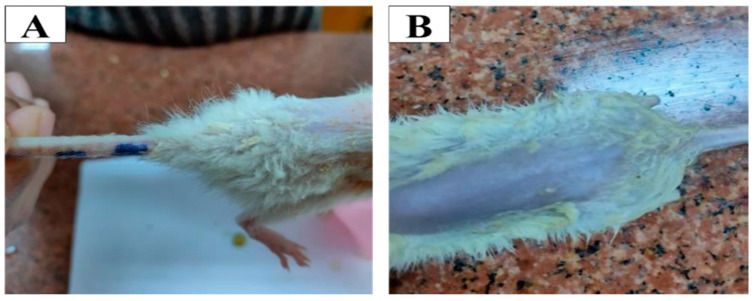
Images showing the rat skin treated with (**A**) the optimized oleogel formulation and (**B**) the lavender oil.

Histopathological examination of groups treated with the optimized oleogel formulation (group II) and lavender oil (group III) displayed no histopathological alterations in different skin layers of the epidermis and dermis upon comparison with the normal untreated skin (group I). Photomicrographs taken for microscopic examination of skin grafts excised from all groups (I, II, and III) showed perfect normal skin features. As shown in Figure 6, the epidermis from outside was consisted of Stratum cornum (black star) formed of keratin filaments, stratum granulosa in between, and then from inside stratum spinosum (yellow star) formed of irregular polygonal cells. The dermis layer was formed of a papillary layer (PL), consisting of loose finer collagen and frequent blood vessels, and a deeper reticular dermal layer (RL) formed of thick collagen fibers. Moreover, the scores were zero as Figure 7 illustrated that there were no signs of edema, redness, or scratches in the examined rats treated with the optimized oleogel and the lavender oil, denoting the absence of any itching. These results indicated that optimized oleogel had good safety and was non-irritant to the skin. These findings could be attributed to the incorporation of lavender oil that has anti-inflammatory, anti-allergic, and wounds and burns healing effects [60,61].

#### 3.7.2. Pharmacodynamic StudyEffect of OLM on Blood Pressure

**Figure 8:**
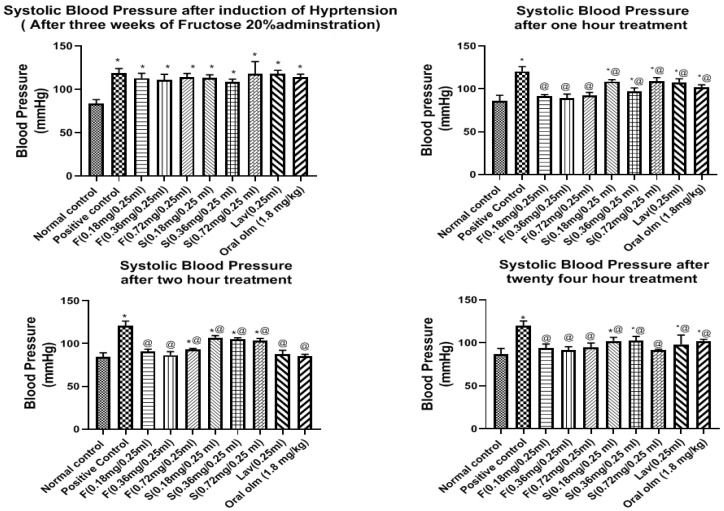
Systolic blood pressure of the ten examined rats’ groups after three weeks of hypertension induction, and after 1, 2, and 24 h of treatment. Presented data are the mean ± SD (n = 3). Abbreviations: F = Transdermal oleogel formulation, S = Transdermal standard gel, Lav= Transdermal Lavender oil (0.25 mL), Oral Olm = Oral Olmesartan Medoxomil market tablet (Angiosartan^®^ Tablets). * Significantly different from the normal control group at respective time interval (*p* < 0.05). @ Significantly different from positive group at respective time interval (*p* < 0.05).

**Figure 9:**
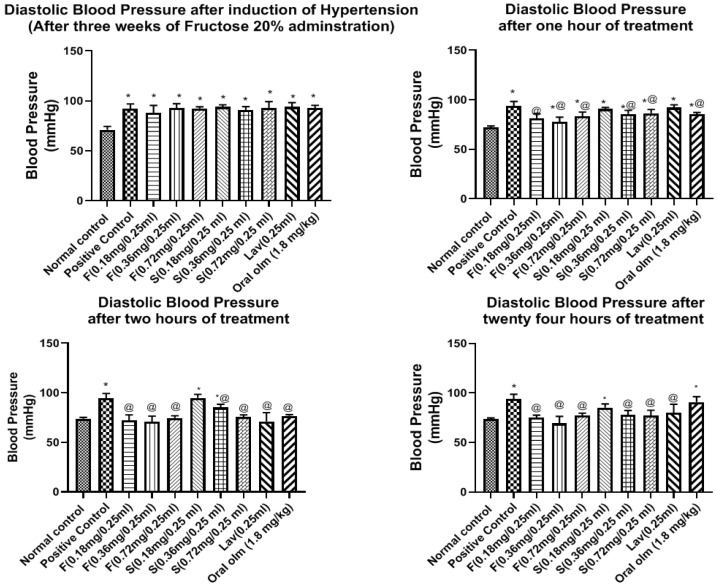
Diastolic blood pressure of the ten examined rats’ groups after three weeks of hypertension induction, and after 1, 2, and 24 h of treatment. Presented data are the mean ± SD (n = 3). Abbreviations: F = Transdermal oleogel formulation, S = Transdermal standard gel, Lav = Transdermal Lavender oil (0.25 mL), Oral Olm = Oral Olmesartan Medoxomil market tablet (Angiosartan^®^ Tablets). * Significantly different from the normal control group at respective time interval (*p* < 0.05). @ Significantly different from positive group at respective time interval (*p* < 0.05).

As illustrated in Figure 8 and Figure 9, the results of BP measured for the in vivo efficacy study revealed that the basal systolic BP for all groups before starting induction of hypertension was 83.46 ± 1.99 mmHg and the diastolic BP was 71.03 ± 1.41 mmHg.

The BP of the normal group was steady throughout the experiment with minimal change in BP of ±2.7 mmHg, and as expected, its sera were free of any treatment traces on HPLC performance.

On the other hand, after three weeks of administration of fructose 20% to rats and supplementation with two subcutaneous dexamethasone injections, there was a significant elevation in the BP of all groups upon comparison with the normal group, as the systolic BP ranged from 108.4 ± 1.41 to 119.9 ± 2.24 mmHg, while the diastolic BP ranged from 88.09 ± 2.99 to 93.9 ± 0.87 mmHg, as shown in Figure 8 and Figure 9, respectively.

Fructose causes hypertension due to endothelial dysfunction leading to impaired relaxation, which is part of metabolic syndrome induced by fructose and this is manifested by hypertension and insulin resistance. Insulin resistance-proposed mechanism of hypertension is the increase in the vascular tone, that is a result of sympathetic nervous system activation, and the elevated production of Endothelin-1 (ET-1), thromboxane A2 (TxA2), and Angiotensin II (Ang II), which finally leads to endothelial dysfunction and vasoconstriction [38]. The administration of dexamethasone also elevated the BP due to increasing insulin resistance. Moreover, it increased the sympathetic tone by potentiating the norepinephrine-induced vasoconstriction, increasing vascular resistance, and decreasing nitric oxide synthesis and cholinergic vasodilator effect. The administration of dexamethasone also exhibited ion flux alterations, cyclooxygenase activation, and Ang I receptors upregulation [62].

Regarding the effect of treatment on the systolic BP, the middle dose of the olmesartan oleogel (0.36 mg/0.25 mL) exhibited the highest and fastest BP lowering effects, as shown in Figure 8. The systolic BP after the first hour of treatment was 91.46 ± 0.79, 89.44 ± 1.83, 92.54 ± 1.36 mmHg for F (0.18, 0.36, 0.72 mg/0.25 mL), 108.5 ± 1.8, 97.46 ± 1.5, 109 ± 1.6 mmHg for S (0.18, 0.36, 0.72 mg/0.25 mL), 107.6 ± 1.7 mmHg for lavender oil (0.25 mL), and 101.8 ± 1.25 mmHg for oral Olmesartan tablet (1.8 mg/kg equivalent to 0.36 mg olm/0.25 mL). After two hours of treatment, the hypotensive effect of the lavender oil and the market tablet started to enhance, where they exhibited systolic BP of 87.20 ± 1.13, 89.54 ± 1.72 mmHg, respectively. In spite of this, the middle dose of the optimized oleogel F (0.36 mg/0.25 mL) was still the best dose that exhibited the strongest BP lowering effects (84.94 ± 1.03). The hypotensive effects of the three dose levels of the oleogel formulations were sustained at the same level for twenty-four hours, as it was 92.71 ± 2.48, 91.2 ± 1.9, and 94.51 ± 2.63 mmHg for F (0.18, 0.36 and 0.72 mg), respectively. On the other hand, the effects of treatment with lavender and oral olmesartan tablet were not sustained as they showed an increase in BP to be 98.19 ± 4.43 and 102.1 ± 0.28 mmHg, respectively.

Regarding the effects of treatment on the diastolic BP, the middle dose of the OLM oleogel (0.36 mg/0.25 mL) also exhibited the highest and fastest BP lowering effects, as shown in Figure 9. The diastolic BP after the first hour of treatment was 81.24 ± 1.86, 77.5 ± 2.04, 83.48 ± 1.65 mmHg for F(0.18, 0.36 and 0.72 mg/0.25 mL), 90.69 ± 0.66, 85.72 ± 1.44, 86.39 ± 1.53 mmHg for S (0.18, 0.36 and 0.72 mg/0.25 mL), 92.24 ± 1.12 mmHg for lavender oil (0.25 mL), and 85.42 ± 0.69 mmHg for oral Olmesartan tablet (1.8 mg/kg equivalent to 0.36 mg/0.25 mL). During the second hour of treatment, the hypotensive effect of oleogel formulations treatment continued to proceed in all groups, as it was 72.29 ± 2.19, 71.87 ± 2.23, 74.58 ± 1.82 mmHg for F (0.18, 0.36 and 0.72 mg), respectively. No improvement was observed for the S (0.18 and 0.36 mg/0.25 mL) groups; however, S (0.72 mg/0.25 mL) showed improvement as the BP became 75.54 ± 0.86 mmHg. As for the lavender oil and the oral OLM tablet groups, they exhibited marked improvement as BP became 72.04 ± 3.61 and 76.52 ± 0.58 mmHg, respectively.

After 24 h, the diastolic BP of groups treated with the three doses of the oleogel formulation was maintained at the same level as the second hour, indicating their sustained effect. On the other hand, the diastolic BP of the lavender and oral Olmesartan tablet treated groups was elevated to 80 ± 3.52 and 90.77 ± 2.23 mmHg, respectively. The hypotensive effect of the three dose levels of the oleogel was significantly different from the untreated group throughout the experiment, unlike oral tablet treatment, where its impact was not considerably different compared to the untreated group after 24 h, as shown in Figure 9.

These findings reveal that the hypotensive effect of the oleogel formulation is rapid, progressive, and sustained for more extended periods when compared to oral OLM tablet treatment. The middle dose of the olmesartan oleogel (0.36 mg/0.25 mL) exhibited the highest and fastest systolic and diastolic BP lowering effects.

The exerted hypotensive effect of lavender oil could be attributed to increasing the serum nitrite level and enhancing the endothelium-independent vasorelaxation [1]. These results are in accordance with Sayorwan et al., who declared that inhalation of lavender oil by healthy human volunteers led to a significant reduction in BP [63].

#### Effect of OLM on Heart Rate

Before starting the induction of hypertension, the HR was 343.2 ± 17.47 beats/minute. The HR of the normal group was steady throughout the experiment with minimal change in HR ± 12 beats/min. As expected, its sera were free of any treatment traces on HPLC performance.

**Figure 10:**
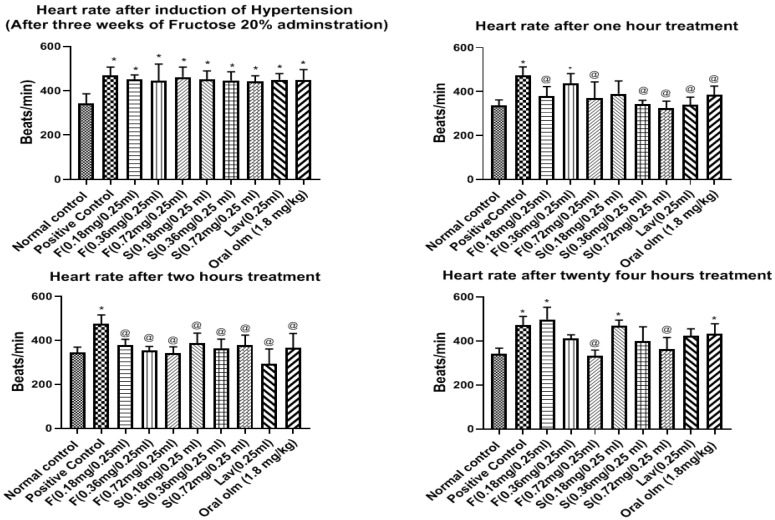
Heart rate of the ten examined rats’ groups after three weeks of hypertension induction, and after 1, 2, and 24 h of treatment. Presented data are the mean ± SD (n = 3). Abbreviations: F = Transdermal oleogel formulation, S = Transdermal standard gel, Lav = Transdermal Lavender oil (0.25 mL), Oral Olm = Oral Olmesartan Medoxomil market tablet (Angiosartan^®^ Tablets). * Significantly different from the normal control group at respective time interval (*p* < 0.05). @ Significantly different from positive group at respective time interval (*p* < 0.05).

On the other hand, subsequent to the hypertension induction in rats, by three weeks of fructose administration and supplementation with two subcutaneous dexamethasone injections, the HR of all the treated groups was significantly more than that of the normal group and ranged from 441.4 ± 10.69 to 461.6 ± 18.53 beats/min, as shown in Figure 10.

The effect of oleogel treatment on HR during the first hour was irregular, but in the second hour, there was an improvement in the impact manifested by bradycardia. This effect followed the dose dependence manner for all treated groups, where the highest impact was of the oleogel formulations F (0.72 mg/0.25 mL) (341.6 ± 11.97), followed by F (0.36 mg/0.25 mL) (346.7 ± 18.2 beats/min), and F (0.18 mg/0.25 mL) (374.9 ± 16.1 beats/min). Oleogel formulations and lavender oil showed the highest effect in decreasing the HR followed by the market tablet and the standard gels.

It is notable that lavender oil had a HR decreasing effect. This could be attributed to the positive impact of lavender oil on the autonomic nervous system, which led to reducing the heart rate. These results are in accordance with those obtained by Kwon et al., who reported that using OLM dramatically elevated the examined rat’s heart rate with reducing their BP, while upon combining OLM with Linalyl acetate (the major constituent of lavender oil), the Olmesartan-induced increase in heart rate was prevented [1]. Moreover, Chien et al. stated that inhalation of lavender oil significantly reduced the heart rate in patients with insomnia by elevating the vagal tone while preserving the sympathetic tone [64]. Therefore, the combination of OLM plus lavender oil can be an effective solution for the treatment of hypertension.

After 24 h, oleogel formulation F (0.72 mg/0.25 mL) showed the highest effect in decreasing the HR (331.6 ± 11.6 beats/min) followed by oleogel formulation F (0.36 mg/0.25 mL) (389.8 ± 17.94 beats/min), indicating their sustained effect compared to oral OLM tablet treatment. The HR of the untreated group (positive control) persisted high throughout the experiment without any reduction, as shown in Figure 10.

Based on the pharmacodynamic results, OLM oleogel formulation F (0.36 mg/0.25 mL) was chosen as the best formulation as it exhibited the highest and fastest systolic and diastolic BP lowering effects besides preventing OLM-induction increase in heart rate. Hence, the OLM concentration of 0.36 mg/mL was chosen for further biochemical and pharmacokinetic studies.

#### 3.7.3. Blood Sampling for Biochemistry

OLM is an angiotensin-II receptor antagonist that binds to angiotensin I and II receptors at the arterioles, thus inhibiting vasoconstriction and lowering blood pressure. The competitive blockage of the angiotensin-II receptor inhibits the aldosterone release from the adrenal cortex, which reduces sodium reabsorption and results in natriuresis, a desirable feature to decrease the elevated blood pressure [65].

Table 3 showed that the Na^+^ level in the sera positive group was significantly higher than that of the normal group. The oleogel formulation F (0.36 mg/0.25 mL) followed by the oral market tablet (1.8 mg/kg equivalent to 0.36 mg/0.25 mL) significantly decreased the Na^+^ levels in sera back to the normal level compared to the positive group (*p*-value < 0.05). The Na^+^ levels in the standard gel (0.36 mg/0.25 mL) and lavender oil (0.25 mL) were significantly higher than those in the normal group, as the Na^+^ level was non-significantly different from that of the positive group (*p*-value > 0.05).

Additionally, one of the critical side effects of oral OLM is hyperkalemia that causes tachycardia [66]. Table 3 showed that the K^+^ level in the group administered the OLM oral market tablet was significantly higher than that of the normal group, while the optimized oleogel F successfully maintained the normal level of K+ in the treated group serum, preventing OLM-induced tachycardia, followed by the lavender oil and the standard gel.

#### 3.7.4. Pharmacokinetic Study

**Figure 11:**
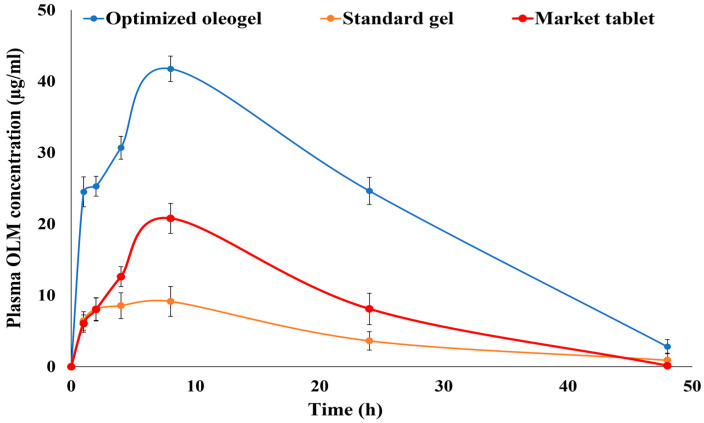
Mean plasma concentrations of olmesartan after a single transdermal application of the optimized oleogel, standard gel, and oral administration of the market product (Angiosartan^®^ Tablets), each equivalent to 0.36 mg drug. Presented data are the mean ± SD (n = 3).

For estimating bioequivalence, the OLM concentration in the plasma of the group treated with oleogel formulation F (0.36 mg/0.25 mL) was compared to that of groups treated with OLM standard gel S (0.36/0.25 mL) and OLM oral market tablet (1.8 mg/kg equivalent to 0.36 mg/0.25 mL). As displayed in Table 4 and Figure 11, the C_max_ value of the optimized oleogel formulation (41.75 ± 6.84 µg/mL) was significantly higher than that of the standard gel (9.14 ± 2.95 µg/mL) and the market product (20.80 ± 5.14 µg/mL) (*p*-value < 0.005). Moreover, the optimized oleogel formula and the standard gel had a significantly higher t_1/2_ and MRT, and significantly lower K_e_ compared to the market tablet (*p*-values < 0.005). These results indicated delivering OLM from both the oleogel and the standard gel with a significantly slower rate compared to the oral market tablet (*p*-value < 0.005). The AUC_0–∞_ of the oleogel formulation showed more than 4.5- and 2.5-folds increase in bioavailability than the standard gel and the market tablet, respectively (*p*-value < 0.005).

The significantly higher bioavailability of the transdermal oleogel formulation than the oral market tablet might be due to the fact that the transdermal route of administration avoided many of the oral administration route’s problems, such as the hepatic first-pass effect and the efflux pumps in the gastrointestinal tract [2,3]. These results are supported by those obtained by Kamran et al. who formulated a transdermal invasomal gel loaded with OLM. They reported that after implanting the in vivo study in Wistar rats, the optimal invasomal gel achieved a 1.15-fold increase in OLM bioavailability compared with the OLM market tablet [2]. They attributed this to ability of the transdermal route to bypass the hepatic metabolism of OLM. These results are also in harmony with that of Albash et al. who formulated transdermal bilosomes loaded with OLM and reported that upon the in vivo kinetic study in albino rabbits, the transdermal optimal bilosomal formulation was found to significantly increase the bioavailability of OLM by more than 2-fold than the oral tablet [5]. They also attributed this to avoiding OLM oral problems via the transdermal route of administration.

On the other hand, the significantly higher relative bioavailability of the transdermal oleogel formulation than the transdermal standard gel could be associated with the incorporation of Tween 20, which increased OLM solubility and absorption and also acted as a permeation enhancer [17,18,19]. This could also be attributed to the incorporation of lavender oil that enhanced OLM solubility, acted as a permeation enhancer, and imparted a lipophilic nature that boosted the drug’s capability of penetrating the skin [13,14]. Moreover, the incorporation of Aerosil provided a bioadhesive oleogel formulation with an appropriate consistency that increased the drug’s contact time on the skin and allowed a more extended absorption period via the skin [55].

## 4. Conclusions

Oleogel formulations were fabricated for the transdermal delivery of OLM using a central composite response surface statistical design. The optimized oleogel formulation showed a significant increase in OLM in vitro release and ex vivo permeation upon comparison with both the drug suspension and gel. Additionally, the histopathological examination assured the tolerability and safety of the optimized oleogel formulation. Moreover, the pharmacodynamic study revealed the superiority of the optimized oleogel formulation, with the middle dose (0.36 mg/0.25 mL), over the market tablet as it maintained both the BP and HR at their normal values for 24 h. The optimized formulation also prevented OLM-induced tachycardia by achieving the best serum electrolyte balance profile. Moreover, the optimized oleogel formulation had a significantly higher bioavailability with more than 4- and 2-folds compared to the standard gel and the oral market tablet, respectively. From these highlighted results, the oleogel formulation could be considered a promising carrier for the transdermal delivery of OLM as it can avoid its oral problems, augment its therapeutic efficacy, decrease its oral side effects, and increase its bioavailability. Further studies are required to assess the therapeutic activity of the optimized oleogel formulation in humans.

## Figures and Tables

**Table 1:** Experimental runs, independent variables, pharmaceutical evaluation, and measured responses of the central composite response surface experimental design for OLM prepared oleogel formulations.

	X_1_: Oil/SAA Ratio	X_2_: Aerosil % (*w/v*)	pH	Drug Content (%)	Viscosity (cps)	Firmness (N)	Compressibility (mJ)	Adhesiveness (mJ)	Fmax (mN)	Wad (µJ)
F1	1	4	5.51 ± 0.13	98.46 ± 3.71	686.4 ± 13.90	42.3 ± 2.98	87.7 ± 4.40	−76.2 ± 5.35	131.6 ± 4.79	176.4 ± 3.96
F2	1	9	5.26 ± 0.15	97.27 ± 5.11	6664 ± 42.48	61.5 ± 4.02	178.2 ± 7.63	−235.6 ± 11.60	539.7 ± 16.11	741.7 ± 20.48
F3	1	14	4.91 ± 0.27	95.51 ± 2.04	17620 ± 79.22	151.8 ± 11.68	308.5 ± 16.81	−230.4 ± 8.97	445.1 ± 21.04	710.2 ± 18.66
F4	5	4	5.17 ± 0.12	99.06 ± 4.95	982.5 ± 37.09	53.1 ± 3.44	113.1 ± 6.04	−119.3 ± 3.41	157.3 ± 8.12	198.9 ± 7.41
F5	5	9	6.20 ± 0.31	97.13 ± 1.84	16670 ± 55.24	129.6 ± 8.92	269.4 ± 18.39	−301.8 ± 15.08	449.1 ± 17.31	507.1 ± 11.38
			6.09 ± 0.33	96.85 ± 3.61	15628 ± 63.28	137 ± 6.87	261.6 ± 21.40	−309 ± 10.42	438 ± 13.56	477.3 ± 9.21
			6.18 ± 0.26	97.22 ± 1.46	16593 ± 80.69	126.2 ± 9.46	257.3 ± 12.43	−295.1 ± 7.14	451.5 ± 20.63	492.1 ± 13.14
F6	5	14	5.73 ± 0.40	94.86 ± 2.01	36670 ± 137.58	213.4 ± 10.83	408 ± 26.68	−226.3 ± 13.64	393.2 ± 14.53	415.6 ± 10.08
F7	9	4	6.11 ± 0.15	96.50 ± 2.83	6948 ± 53.90	84.9 ± 22.64	172.1 ± 4.29	−203.5 ± 6.77	314.1 ± 6.16	280.9 ± 7.65
F8	9	9	4.99 ± 0.31	98.34 ± 1.45	29370 ± 94.56	186.3 ± 4.91	324.4 ± 15.75	−390.1 ± 11.82	460.8 ± 23.74	547.5 ± 13.89
F9	9	14	5.87 ± 0.21	95.94 ± 3.42	48620 ± 183.65	221.7 ± 7.64	427.6 ± 10.36	−349.6 ± 12.16	415.6 ± 15.73	508.2 ± 12.09

Presented data are the mean ± SD (n = 3).

**Table 2:** Output data of the central composite response surface statistical design with the predicted and observed values of the optimized oleogel formulation.

Responses	Viscosity (cps)	Firmness (N)	Compressibility (mJ)	Adhesiveness (mJ)	Fmax (mN)	Wad (µJ)
Minimum	686.4 ± 17.90	42.3 ± 2.98	87.7 ± 4.40	−76.2 to ± 5.35	131.6 ± 4.79	176.4 ± 3.96
Maximum	48,620 ± 183.65	221.7 ± 7.64	427.6 ± 10.36	−390.1 ± 11.82	539.7 ± 16.11	741.7 ± 20.48
Model	2 FI	Linear	Linear	Quadratic	Quadratic	Quadratic
F value	170.78	61.05	189.48	52.24	18.77	18.33
*p*-value	<0.0001	<0.0001	<0.0001	0.0003	0.0030	0.0031
Adequate precision	40.83	23.93	40.29	23.51	11.83	13.33
Adjusted R^2^	0.981	0.923	0.974	0.962	0.898	0.896
Predicted R^2^	0.951	0.863	0.954	0.840	0.469	0.475
R^2^	0.986	0.940	0.980	0.981	0.949	0.948
Significant factors	X_1_, X_2_, and X_1_·X_2_	X_1_ and X_2_	X_1_ and X_2_	X_1_, X_2_, X_2_^2^	X_2_, X_1_·X_2_, X_2_^2^	X_2_, X_1_·X_2_, X_1_^2^, X_2_^2^
Observed values of optimal oleogel	10,769.4	113.19	229.97	−251.63	532.12	730.86
Predicted values of optimal oleogel	10,852.9	109.43	236.91	−266.72	520.58	734.85

**Table 3:** Effect of effective dose of OLM oleogel formulation F (0.36 mg/0.25 mL) on serum level of Na^+^ and K^+^ vs. OLM standard gel S (0.36 mg/0.25 mL), lavender oil, and oral OLM market tablet (1.8 mg/kg equivalent to 0.36 mg/0.25 mL).

Group Parameter	Normal Control	Positive Control	F (0.36 mg/0.25 mL)	S (0.36 mg/0.25 mL)	Lavender Oil (0.25 mL)	Oral OLM (1.8 mg/kg)
Na^+^	132.3 ± 1.2	146 ± 0.57	134 ± 0.7	144 ± 0.84	145 ± 0.54	137.7 ± 0.88
K^+^	4.2 ± 0.1	3.33 ± 0.08	4.4 ± 0.17	4.7 ± 0.11	4.5 ± 0.06	5.5 ± 0.23

F = Transdermal OLM oleogel formulation, S = Transdermal OLM Standard gel, Oral OLM = Oral OLM market tablet. Each value represents a mean of serum level of Na^+^ or K^+^ ± SD (n = 6 rats).

**Table 4:** Drug pharmacokinetic parameters of OLM after the transdermal application of the optimized oleogel formulation (0.36 mg/0.25 mL), standard gel (0.36 mg/0.25 mL), and oral administration of the market tablet (1.8 mg/kg equivalent to 0.36 mg/0.25 mL).

Pharmacokinetics Parameter	Optimized Oleogel	Treatment (Mean ± SD)	
		Standard Gel	Market Tablet
C_max_ (µg/mL) ^a^	41.75 ± 6.84	9.14 ± 2.95	20.80 ± 5.14
AUC_0-48_ (ng.h/mL) ^a^	1098.50 ± 72.41	218.85 ± 35.82	427.56 ± 57.53
AUC_0-∞_ (ng.h/mL) ^a^	1145.19 ± 84.28	234.38 ± 41.13	429.00 ± 68.91
t_max_ (h) ^a^	8 ± 0.00	8 ± 0.00	8 ± 0.00
t_1/2_ (h) ^a^	9.99 ± 1.47	10.65 ± 0.94	5.39 ± 1.26
K (l/h) ^a^	0.07 ± 0.01	0.06 ± 0.00	0.13 ± 0.01
MRT ^a^	17.60 ± 6.84	17.63 ± 7.11	13.73 ± 4.92
% Relative bioavailability (%RB)	488.60% (compared to the standard gel) 266.94% (compared to the market tablet)	-	-

^a^ Presented data are the mean ± SD (n = 6).

## Data Availability

Not applicable.

## Supplementary Materials

The following supporting information can be downloaded at: https://www.mdpi.com/article/10.3390/pharmaceutics15041083/s1, Figure S1: Effect of interaction between the two studied factors of O/S ratio (X_1_) and Aerosil % (X_2_) on Viscosity, Figure S2: Effect of interaction between the two studied factors of O/S ratio (X_1_) and Aerosil % (X_2_) on Fmax, Figure S3: Effect of interaction between the two studied factors of O/S ratio (X_1_) and Aerosil % (X_2_) on Wad.
