# Supplementary material for: Development of Transdermal Oleogel Containing Olmesartan Medoxomil: Statistical Optimization and Pharmacological Evaluation
Source: Pharmaceutics. 2023 Mar 28;15(4):1083. doi: 10.3390/pharmaceutics15041083 (PMC10146305; doi:10.3390/pharmaceutics15041083)
Supplement: Supplementary file 1 [file pharmaceutics-15-01083-s001.zip › Figures caption for supplementary figures.pdf]

Supplementary Figure S1. Effect of interaction between the two studied factors of O/S ratio (X_1) and Aerosil % (X_2) on Viscosity.

Supplementary Figure S2. Effect of interaction between the two studied factors of O/S ratio (X_1) and Aerosil % (X_2) on Fmax.

Supplementary Figure S3. Effect of interaction between the two studied factors of O/S ratio (X_1) and Aerosil % (X_2) on Wad.
