# Supplementary figures and images for: Development of Transdermal Oleogel Containing Olmesartan Medoxomil: Statistical Optimization and Pharmacological Evaluation
Source: Pharmaceutics. 2023 Mar 28;15(4):1083. doi: 10.3390/pharmaceutics15041083 (PMC10146305; doi:10.3390/pharmaceutics15041083)

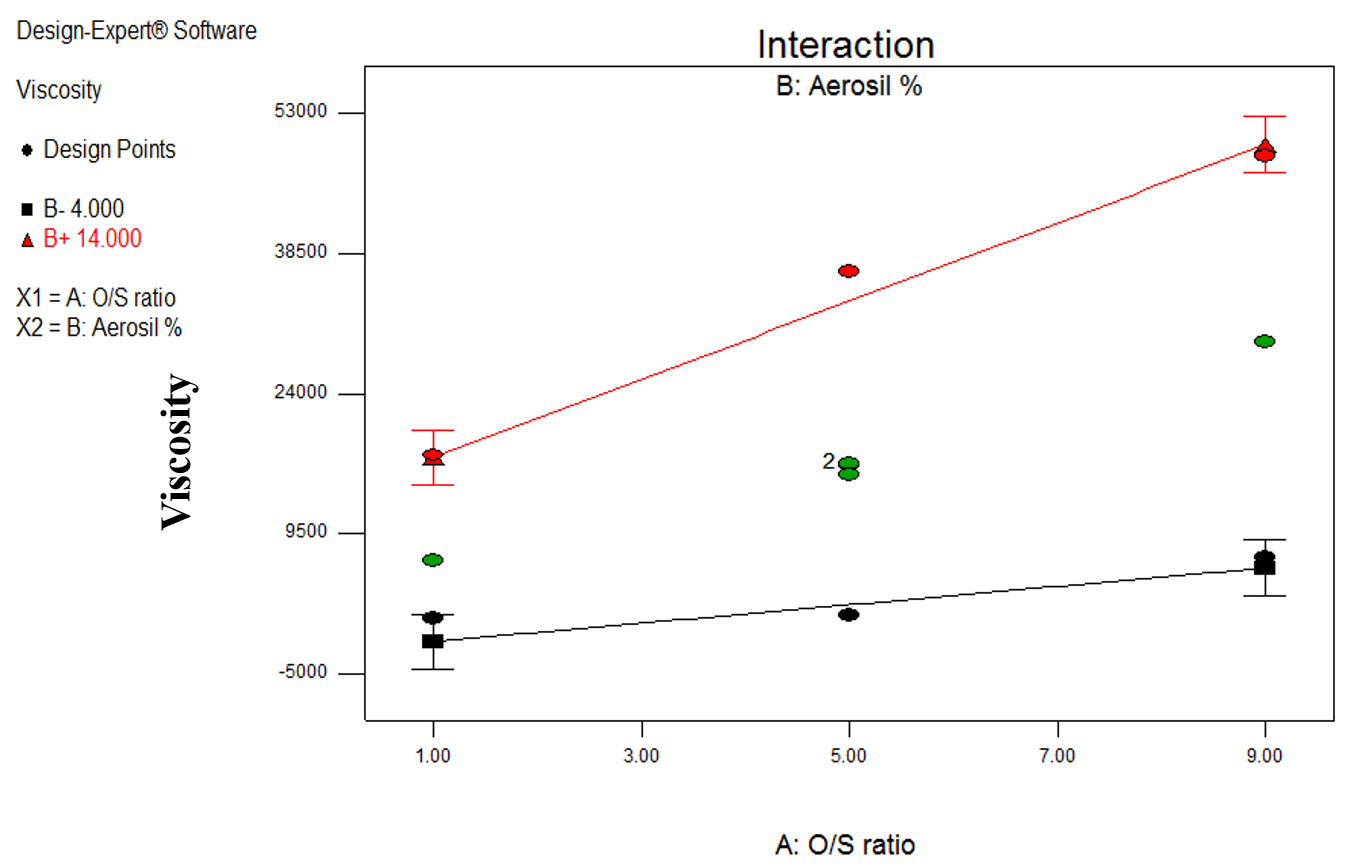

Supplement: Supplementary file 1 [file pharmaceutics-15-01083-s001.zip › Supplementary Figure S1.tiff]

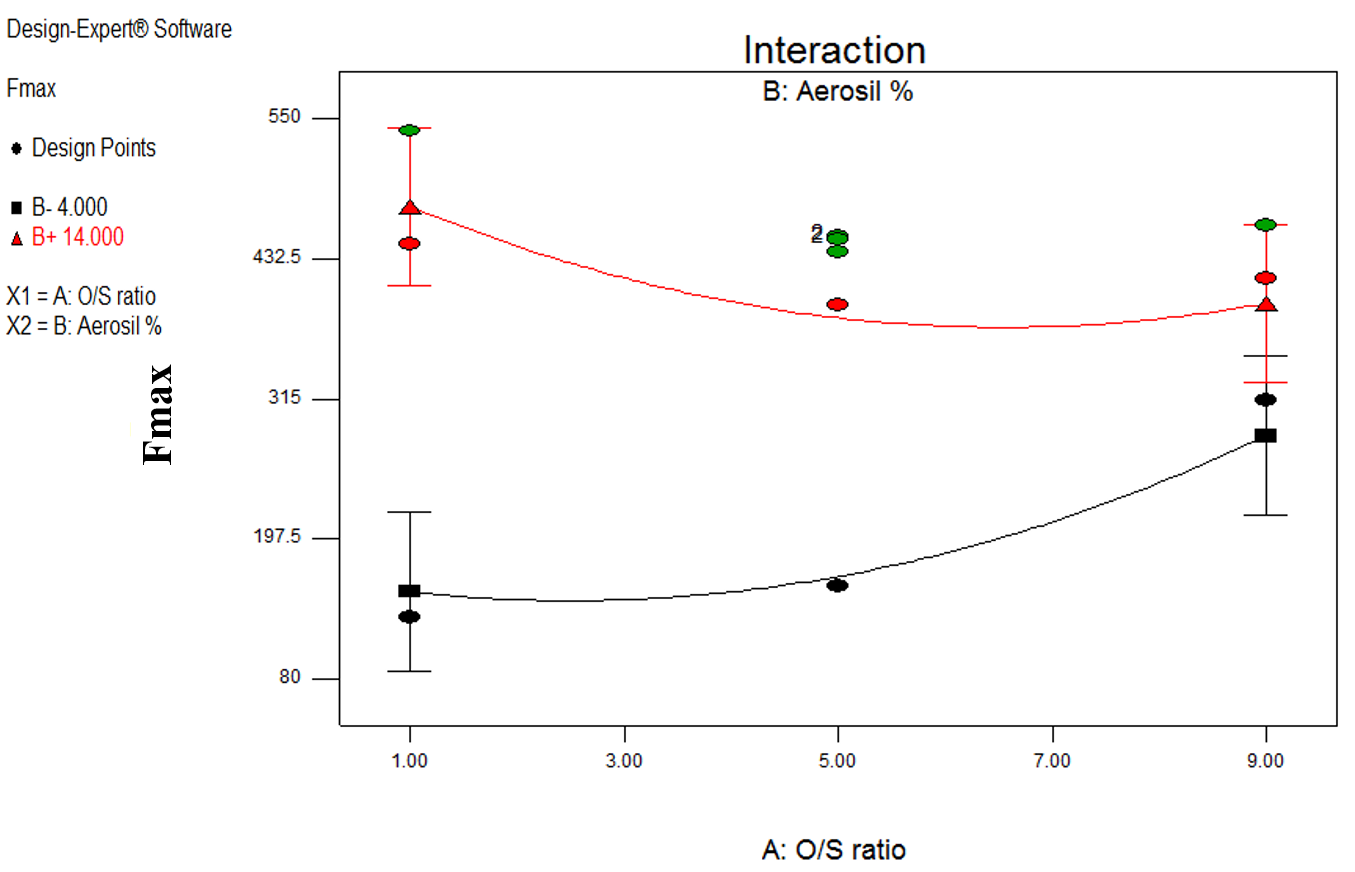

Supplement: Supplementary file 1 [file pharmaceutics-15-01083-s001.zip › Supplementary Figure S2.tiff]

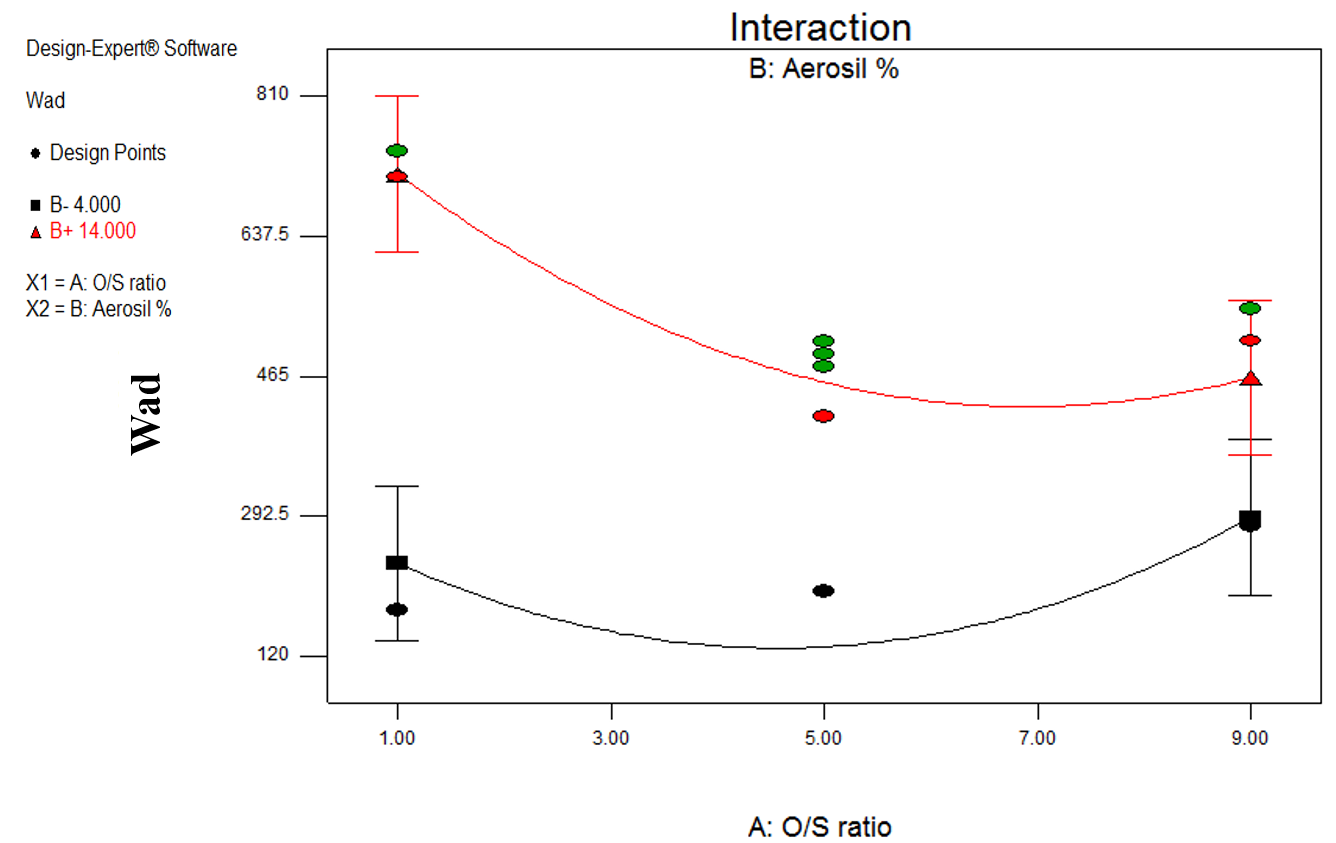

Supplement: Supplementary file 1 [file pharmaceutics-15-01083-s001.zip › Supplementary Figure S3.tiff]
